# Tumoral parkinsonism—Parkinsonism secondary to brain tumors, paraneoplastic syndromes, intracranial malformations, or oncological intervention, and the effect of dopaminergic treatment

**DOI:** 10.1002/brb3.3151

**Published:** 2023-07-11

**Authors:** Gustav Cedergren Weber, Jonathan Timpka, Anna Rydelius, Johan Bengzon, Per Odin

**Affiliations:** ^1^ Division of Neurology, Department of Clinical Sciences Lund, Faculty of Medicine Lund University Lund Sweden; ^2^ Department of Neurology, Rehabilitation Medicine, Memory and Geriatrics Skåne University Hospital Lund Sweden; ^3^ Division of Neurosurgery, Department of Clinical Sciences, Kamprad laboratory Lund University Lund Sweden; ^4^ Department of Neurosurgery Skåne University Hospital Lund Sweden

**Keywords:** brain neoplasm, levodopa, meningioma, nonmotor symptoms, secondary parkinsonism

## Abstract

**Introduction:**

Secondary tumoral parkinsonism is a rare phenomenon that develops as a direct or indirect result of brain neoplasms or related conditions.

**Objectives:**

The first objective was to explore to what extent brain neoplasms, cavernomas, cysts, paraneoplastic syndromes (PNSs), and oncological treatment methods cause parkinsonism. The second objective was to investigate the effect of dopaminergic therapy on the symptomatology in patients with tumoral parkinsonism.

**Methods:**

A systematic literature review was conducted in the databases PubMed and Embase. Search terms like “secondary parkinsonism,” “astrocytoma,” and “cranial irradiation” were used. Articles fulfilling inclusion criteria were included in the review.

**Results:**

Out of 316 identified articles from the defined database search strategies, 56 were included in the detailed review. The studies, which were mostly case reports, provided research concerning tumoral parkinsonism and related conditions. It was found that various types of primary brain tumors, such as astrocytoma and meningioma, and more seldom brain metastases, can cause tumoral parkinsonism. Parkinsonism secondary to PNSs, cavernomas, cysts, as well as oncological treatments was reported. Twenty‐five of the 56 included studies had tried initiating dopaminergic therapy, and of these 44% reported no, 48% low to moderate, and 8% excellent effect on motor symptomatology.

**Conclusion:**

Brain neoplasms, PNSs, certain intracranial malformations, and oncological treatments can cause parkinsonism. Dopaminergic therapy has relatively benign side effects and may relieve motor and nonmotor symptomatology in patients with tumoral parkinsonism. Dopaminergic therapy, particularly levodopa, should therefore be considered in patients with tumoral parkinsonism.

## Graphical abstract

Brain neoplasms, paraneoplastic syndromes, and oncological treatments can cause parkinsonism. Dopaminergic therapy has relatively benign side effects and may relieve motor and nonmotor symptomatology in patients with tumoral parkinsonism. Dopaminergic therapy, particularly levodopa, should therefore be considered in patients with tumoral parkinsonism.

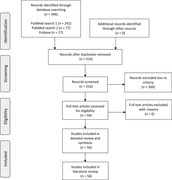

## 1 INTRODUCTION

Secondary parkinsonism caused by tumors, or in short tumoral parkinsonism, is defined as parkinsonism developing as a direct or indirect result of tumors, most frequently through mesencephalic infiltration or compression (Bhatoe, 1999). However, parkinsonian symptomatology in patients with brain tumors is rarely described. In a prospective evaluation, eight out of 907 patients with supratentorial brain tumors were found to suffer from some type of secondary parkinsonism—indicating a prevalence of 0.3% (Krauss et al., 1995). The most common localization of these neoplasms is supratentorial. Tumor entities are most often meningioma, followed by glioma (usually astrocytoma), central nervous system (CNS) lymphoma, and craniopharyngioma (Höllerhage, 2019). Various types of brain cysts, cavernomas, and brain metastases have also been reported to cause secondary parkinsonism (Chang et al., 2000; Hortelano et al., 2010; Ishihara et al., 2011). Other relevant medical causes in the context of a broader definition of tumoral parkinsonism include paraneoplastic syndromes (PNSs) (Golbe et al., 1989; Topcular et al., 2013) and iatrogenic oncological parkinsonism (Franchino et al., 2019; Markman et al., 1985; Skiming et al., 2003; Wenning et al., 1999). Surgical removal or complete resection of the tumor generally has a good effect on parkinsonian symptomatology, often resulting in marked improvement or complete reversal of symptoms. As for dopaminergic therapy, the general opinion is that it has little to no effect, but different articles have reported conflicting results. No controlled trials have been performed (Höllerhage, 2019).

This systematic synthesis of the literature was motivated by the clinical experience of six patients suffering from parkinsonism secondary to different types of brain neoplasms (Table 1). Interestingly, all these cases improved significantly after initiation of dopaminergic therapy. These findings contrast the current oncological and neurological consensus (Höllerhage, 2019). As there were no current compilations of data on this topic, this justified a crude analysis of outcome of dopaminergic therapy on tumoral parkinsonism patients, primarily collected from case reports. The review highlights findings and advances made in this relatively underexplored field. Among the questions to be answered are: which different brain neoplasms can cause tumoral parkinsonism, and what common features are they characterized by? To which degree and how do PNSs and oncological treatment cause parkinsonism? Finally, what do published studies indicate regarding the effects of dopaminergic therapy on symptomatology in patients with tumoral parkinsonism?

**TABLE 1:** Patients with parkinsonism secondary to brain neoplasms that served as the primary motivation for conducting the review.

Patient	1	2	3	4
Medical history regarding underlying tumor causing secondary parkinsonism.	Oligodendroglioma II, left frontal lobe. Resection: years later became malignant—frontal lobe resection, cytostatic, proton radiation. Epilepsy as complication.	Astrocytoma II, left frontotemporal area. Resection. Years later: recurrent tumor, treated with resection and radiation.	Malignant mandibular tumor. Resection, radiotherapy.	Cerebral lymphoma. Extensive treatment including stem cell support.
Age of onset (parkinsonism), sex	50–59, male	50–59, female	50–59, female	60–69, male
Symptoms	Right‐side parkinsonism: tremor, hypokinesia. Nonmotor: day‐time tiredness.	Slight bradykinesia, pronounced fatigue, restless legs (RLS)	After radiotherapy gait difficulties, small steps, strong tendency to freezing, difficult to walk.	After lymphoma treatment fatigue, anhedonia, apathy, mild depression, reduced cognition, tremor upper extremity (postural and action), generalized hypokinesia, no rigidity
Neuroimaging of parkinsonism correlates	DAT‐scan: pathology, left side	DAT‐scan: not performed	DAT‐scan: not performed MRI: diffuse signal changes in large parts of the brain	DAT‐scan: borderline FDG‐PET: frontal reduction both sides
Treatment	Levodopa responsive. I. Levodopa–carbidopa 300 mg/d + pramipexole 1.05 (clear improvement, only micrographia remains) II.. Levodopa–carbidopa 1000 mg/day, safinamide added, pramipexole remains (good control, no fluctuation, no dyskinesia) III. Amantadine added for daytime sleepiness—partial effect IV. Patient deceased from tumor	Pramipexole responsive. I. SSRI, Mirtazapine, Saroten were tried against fatigue: no effect. II. Bromocriptine 7.5 mg: fatigue and RLS improved, stopped because of side effects III. Ropinirole: partial effect on fatigue and RLS. IV. Rotigotine 4 mg: good effect on fatigue and RLS, but felt “sped up” V. Pramipexol, slowly increased to 2.1 mg: excellent effect on fatigue and RLS, later changed back to 1.31 mg since patient felt “sped up”	Levodopa responsive. I. Levodopa–carbidopa 100/25 × 3 po, normalized gait, no parkinsonian symptoms, no side effects.	Levodopa responsive. I. Citalopram, Mirtazapine no effect II. Levodopa–carbidopa 100/25 1.5 × 3 – tremor improves, neuropsychiatric symptoms still unchanged

*Note*: Out of six treated patients in total, four patients signed a written consent to share their medical history, one patient declined to participate, and one patient was untraceable. Medical history related to oncological treatment of underlying tumor, investigation of neurological deficits, as well as symptomatic treatment of secondary parkinsonism is presented. All six cases, including the two with unspecified medical history, improved significantly after initiation of dopaminergic therapy.

The aim was to make this review useful in a practical clinical setting, by categorizing relevant data according to tumoral entity, to facilitate finding information about a specific tumor entity or phenomenon of interest.

## 2 METHODS

### 2.1 Search strategy

The search for articles in this review was performed through the medical databases PubMed and Embase. Two separate searches were carried out in PubMed and one in Embase. Examples of terms included in these searches are “secondary parkinsonism,” “astrocytoma,” and “cranial irradiation.” Synonyms and terms related to all search terms were allowed and used. The search strategy required the terms to be found in either the title or abstract of the articles, or to be subject headings such as MeSH terms or Emtree terms of the article. For a complete presentation of the database search, specifying all strategies and terms utilized in this literature review, please see Table 2.

**TABLE 2:** PubMed and Embase search strategy.

Database: PubMed date: July 6, 2022	Search terms	Number of matches	Included in review
#1	Secondary Parkinson Disease [MeSH Terms] OR Secondary Parkinsonism [MeSH Terms] OR Secondary Parkinsonism [T/A]		
#2	Brain Tumor [MeSH Terms] OR Brain Neoplasm [MeSH Terms] OR Brain Cancer [MeSH Terms] OR Brain Neoplasm, Malignant [MeSH Terms] OR Brain Tumor [T/A] OR Brain Neoplasm [T/A] OR Brain Cancer [T/A] OR Brain Metastasis [T/A] OR Meningioma [MeSH Terms] OR Astrocytoma [MeSH Terms] OR Meningioma [T/A] OR Astrocytoma [T/A] OR Arachnoid Cyst [MeSH Terms] OR Arachnoid Cyst, Intracranial [MeSH Terms] OR Cranial Irradiation [MeSH Terms] OR Chemotherapy [T/A] OR Radiation [MeSH Terms]		
#3	#1 AND #2	77	31
#1	Secondary parkinsonism cancer tumor [Free text]	242	14
#1	Secondary Parkinsonism [exp] OR Secondary Parkinsonism [ti,ab,kw]		
#2	Brain Cancer [exp] OR Brain Cancer [ti,ab,kw] OR Brain Tumor [exp] Brain Tumor [ti,ab,kw] OR Astrocytoma [exp] OR Astrocytoma [ti,ab,kw] OR Meningioma [exp] OR Meningioma [ti,ab,kw] OR Arachnoid Cyst [exp] OR Arachnoid Cyst [ti,ab,kw] OR Brain Metastasis [exp] OR Brain Metastasis [ti,ab,kw] OR Chemotherapy [exp] OR Chemotherapy [ti,ab,kw] OR Radiotherapy [exp] OR Radiotherapy [ti,ab,kw]		
#3	#1 AND #2	27	11

*Note*: Two separate searches were carried out in PubMed and one in Embase. Articles identified with this systematic search approach were screened for eligibility before inclusion in the study.

Abbreviations: exp, exploded—Emtree term; T/A, Title/Abstract; ti,ab,kw, Title, Abstract, Author Keywords.

### 2.2 Abstract inspection

The articles collected from the database search were inspected by reading the title, abstract, and keywords.

Full‐text reading was carried out on articles fulfilling the required inclusion criteria:

- The study had to focus on a subtype or aspect of parkinsonism secondary to
  ○ Brain tumors OR
  ○ PNSs OR
  ○ Nontumoral intracranial malformations OR
  ○ Oncological intervention.
- The language of the article must be English.
- Appropriate ethical considerations regarding informed consent, risk of harm, confidentiality and anonymity, and conflict of interest.

The exclusion criteria were as follows: original research must be presented, and review articles were excluded.

### 2.3 Full‐text reading and detailed review

**FIGURE 1:**
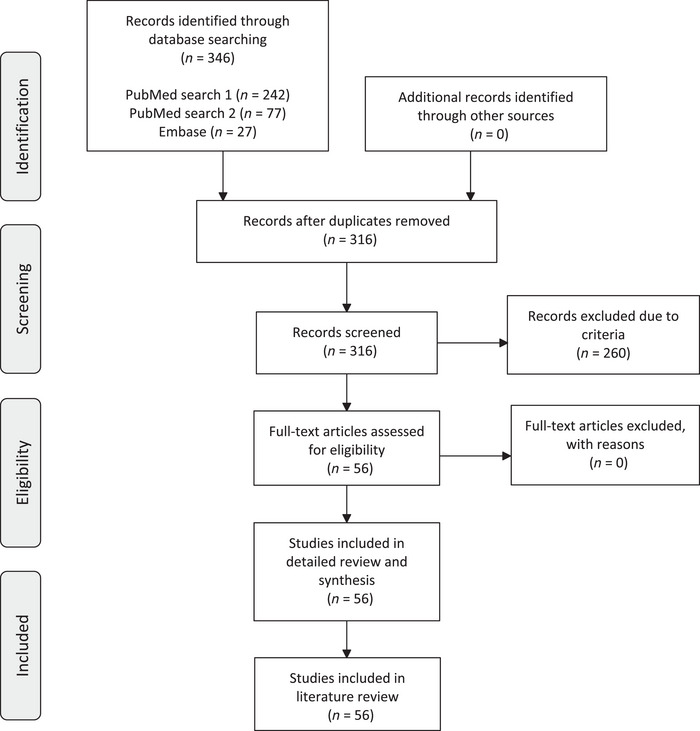
PRISMA 2009 flow diagram of the article selection for the systematic review.

Articles aligning with abovementioned criteria were read in full. New articles discovered in the reference list of other articles or by specific searches could also be further investigated and added to full‐text review. However, these types of articles were reported as a separate category in the flow diagram presented in Figure 1.

Selected articles were subject to thorough review. Hence, data and material from reviewed studies about secondary tumoral parkinsonism and related brain neoplasms, PNSs, intracranial malformations, oncological treatment methods, or the effect of dopaminergic treatment were collected. For a complete flow diagram illustrating the article selection, see Figure 1.

## 3 RESULTS

### 3.1 Primary CNS tumors

#### 3.1.1 Meningioma

Based on the number of case reports in the literature, the most common type of CNS tumor to be reported to cause parkinsonism is meningioma (Höllerhage, 2019), also being the second most common type of tumor in the CNS. However, due to insufficient epidemiologic data, it is impossible to fairly assess the exact incidence of meningiomas, as well as other tumor types, resulting in parkinsonism. Saleh et al. reported a 59‐year‐old female with left‐sided tremor in the hand and foot, atypical bilateral intention tremor, bradykinesia, and several other signs of parkinsonism. Magnetic resonance imaging (MRI) confirmed a meningioma in the right frontal area, compressing the right frontal lobe and nucleus lenticularis. The study concluded that it is important to be suspicious of secondary parkinsonism, and even more so if the patient presents with signs less consistent with idiopathic Parkinson's disease (Saleh et al., 2021). There are numerous examples in the literature of similar cases, presenting with parkinsonism and a meningioma subsequently suspected by neuroradiological examination (Al‐Janabi et al., 2019; Kim et al., 2014; Kleib et al., 2016; McMahan & Galvez, 2015; Okada et al., 1983; Saleem et al., 2000). Miyagi et al. presented a patient with left‐sided hemiparkinsonism caused by a falx meningioma, compressing the right supplementary motor area. The meningioma was successfully removed, and symptoms were reversed. Miyagi et al. (1993) concluded that this case may have been caused by impaired synaptic function of the entire striatum, resulting in contralateral hemiparkinsonism, without a direct impairment on the striatal presynaptic dopaminergic nerve terminals. Although the other studies did not present such thorough imaging workups, this study might suggest that parkinsonism can be the result of either direct pressure from the tumor or a metabolic dysfunction.

#### 3.1.2 Glioma

The second most common type of brain tumor to be reported to cause parkinsonism is glioma, predominantly of the subtype astrocytoma (Höllerhage, 2019). Ho et al. reviewed the medical history of a 60‐year‐old man who developed right‐sided hemiparkinsonism, most notably resting tremor and bradykinesia. Initially, the patient was thought to suffer from idiopathic Parkinson's and was consequently treated with levodopa. Since this had no effect, MRI was conducted for further investigation, revealing an infiltrative astrocytoma spanning from the left mesial temporal lobe to the left basal ganglion and insula. Reversal of initial symptoms was observed after resection, but an ipsilateral hemiparesis presented postoperatively. The histopathological diagnosis was high‐grade astrocytoma (Ho et al., 2008). Others have reported similar symptomatology as a result of corpus callosum astrocytoma (Arcaya Navarro et al., 1986) and brain stem cystic astrocytoma (Cicarelli et al., 1999). Choi et al. presented a patient with an astrocytoma in the third ventricle and hypothalamus, where the major clinical manifestation was mild parkinsonism. This is an unusual case, since it is more common that tumoral parkinsonism is caused by compression or distortion of the basal ganglia from distant intracranial lesions, rather than actual midbrain infiltration. Surgery revealing a diffuse astrocytoma grade II, implantation of a ventriculoperitoneal shunt, and radiotherapy resulted in complete recovery of parkinsonism (Choi et al., 2012). A similar case of a thalamic butterfly astrocytoma (anaplastic astrocytoma grade III) resulting in parkinsonism was reported by Wächter et al. The clinical response to dopaminergic therapy was moderate (Wächter et al., 2011). Researchers from all cited astrocytoma studies assert in their conclusion that one must perform a neuroradiological exam of patients with parkinsonism to exclude potentially treatable structural causes, such as astrocytomas or other neoplastic lesions (Arcaya Navarro et al., 1986; Choi et al., 2012; Cicarelli et al., 1999; Ho et al., 2008; Wächter et al., 2011). Other types of glioma reported to cause parkinsonian symptomatology include oligodendroglioma (de Sèze et al., 1998), glioblastoma (Ruiz‐Escribano Menchen et al., 2020), corpus callosum glioma (Pall & Williams, 1987), and low‐grade glioma (Straube & Sigel, 1988).

#### 3.1.3 CNS lymphoma

Primary CNS lymphoma is a rare disease, primarily affecting older people, and secondary parkinsonism due to CNS lymphoma is extraordinarily rare (Okano et al., 2021). Okano et al. described a 75‐year‐old male who had several lesions in the thalamic and surrounding white matter regions, exhibiting classic signs of parkinsonism. Stereotaxic biopsy was performed, and after confirming tumor‐associated parkinsonism, treatment with corticosteroids and chemotherapeutic agents had an excellent effect, resulting in complete reversal of the neoplasm and parkinsonian symptoms after 8 months (Okano et al., 2021). Gherardi et al. reported a primary CNS large‐cell B‐lymphoma with monoclonal lambda light chains, where the patient presented with typical parkinsonian symptomatology. The initial response to dopaminergic therapy was good, but soon faded. Since neuronal loss, extraneuronal pigment and neuronophagia‐like nodules were observed, and tumoral mass effect was excluded. Gherardi et al. hypothesized that potential neurotoxic effects of the lymphoma could have caused the symptoms of parkinsonism (Gherardi et al., 1985). Kirkedal et al. explored a 73‐year‐old female who exhibited symptoms of right‐sided hemiparkinsonism. MRI and cerebrospinal fluid (CSF) analysis indicated primary CNS diffuse B‐cell lymphoma, and the patient was therefore treated according to standard CNS lymphoma protocol, resulting in partial recovery of the symptomatology and almost complete tumoral regression after 9 months. Kirkedal et al. concluded that MRI should be the firsthand neuroradiological tool if one suspects tumoral parkinsonism, since CNS lymphomas and other neuronal neoplasms usually admit contrast, unlike ischemic changes seen in the differential diagnosis vascular parkinsonism (Kirkedal et al., 2022).

#### 3.1.4 Other types of primary brain tumors

Theoretically, any brain tumor can cause secondary parkinsonism as a result of distant mass effect and metabolic and vascular disturbances (Bhatoe, 1999). Dolendo et al. reported a 14‐year‐old male with immature teratoma in the pineal gland exhibiting parkinsonism. A ventriculoperitoneal shunt improved the hydrocephalus, but dopaminergic therapy did not improve parkinsonian symptoms. Symptoms did improve after reprogramming the shunt. Despite numerous excisions and chemotherapy, the tumor continued to enlarge, and the patient was assessed to suffer from parkinsonism secondary to growing teratoma syndrome (Dolendo et al., 2003). Biochemical studies on a patient with craniopharyngioma similarly suggested the atypical tumor as the cause of parkinsonism (García de Y'ebenes et al., 1982). Parkinsonism in children has been described in two cases of mesencephalic tumors (Pohle & Krauss, 1999).

### 3.2 Intracranial malformations

#### 3.2.1 Cyst

Cysts are capsules filled with tissue, fluid, or air that rarely develop into cancer or metastasize. There are three types of cysts that have been reported to give rise to secondary parkinsonism: arachnoid/leptomeningeal cysts, pineal gland cysts, and epidermoid cysts. Wimmer et al. described a 55‐year‐old male patient with multiple arachnoid cysts spanning from the middle to the posterior fossa, who exhibited hemiparkinsonism (Wimmer et al., 2020). Tiple et al. investigated a 42‐year‐old female with an arachnoid cyst in the frontal lobe and almost identical symptomatology to the case presented by Wimmer et al., although this latter patient also suffered from nonmotor symptoms, most notably depression (Tiple et al., 2010). In both cases, surgery of the cyst resulted in rapid clinical improvement of the patients (Tiple et al., 2010; Wimmer et al., 2020). Pineal gland cysts are quite common and are found in 1%–4% of patients undergoing an MRI. They usually remain asymptomatic. Secondary parkinsonism is a rare clinical presentation. Sarkiss et al. is the only group, as far as the authors are aware, to report hemiparkinsonism secondary to an epidermoid cyst. A 30‐year‐old male presented with unilaterally left‐sided arm tremor, rigidity and bradykinesia, hand weakness, and facial droop. Craniotomy and gross total resection resulted in complete reversal of symptoms (Sarkiss et al., 2019). All described cyst cases exhibited unilateral hemiparkinsonism and improved rapidly after surgical intervention.

#### 3.2.2 Cavernoma

Cavernoma, also known as cavernous angioma or cerebral cavernous malformation, are usually found in the brain and spinal cord. Vhora et al. reported a 55‐year‐old female with a cavernoma in the pineal region, presenting with parkinsonism. The authors speculate that compression of the posterior thalamus and upper mesencephalon with congestion of the deep venous system could have resulted in vascular pathology in the nigro‐striate‐pallidal system. Resection resulted in gradual clinical improvement (Vhora et al., 2001). Ertan et al. investigated a 56‐year‐old male with a cavernoma located in the basal ganglia, who exhibited tremor and bradykinesia. The patient received levodopa, with no effect even at high doses. Chronic compression and hemorrhage were suggested as possible pathological explanations for tumoral parkinsonism in this particular case (Ertan et al., 2005). Alp et al. described an almost identical case with regard to cavernoma localization, clinical presentation, and attitude towards treatment, and similarly argued for chronic compression as an important pathophysiological factor (Alp et al., 2009). Modreanu et al. discussed a 75‐year‐old female with a 9‐year history of nonprogressive hemiparkinsonism and apathy secondary to a cavernoma in the basal ganglia. The authors highlighted the difficulties of differentiating idiopathic and secondary parkinsonism, since dopamine transporter imaging can be pathological in both cases. However, nonprogression, negative levodopa and apomorphine tests, static clinical presentation after levodopa discontinuation, stable hemiparkinsonism across time, and negative transcranial brainstem ultrasound all point in the direction of secondary parkinsonism due to cavernoma rather than idiopathic Parkinson's disease (PD) (Modreanu et al., 2020).

### 3.3 Tumors of extracranial origin

#### 3.3.1 Brain metastasis

Brain metastases can originate from a wide range of neoplasms, but most derive from melanoma, lung, breast, colon, or kidney cancer. Ishihara et al. reported a 68‐year‐old male with CNS metastases of natural killer/T‐cell lymphoma (NKTL) who manifested with pure akinesia, without the typical parkinsonian rigidity and tremor. The patient additionally suffered from short steps, a forward flexion posture, festination, postural instability, facial hypomimia, and micrographia. MRI with diffusion‐weighted imaging and CSF cytology confirmed NKTL in the dorsal brainstem, periventricular white matter, and cerebellum, later on spreading to the basal ganglia and infratentorial regions. Levodopa and intrathecal methotrexate had no clinical effect. Second‐line treatment with methotrexate pulse therapy and cytosine arabinoside was initiated and resulted in markedly improved clinical picture and reduced the tumoral burden (Ishihara et al., 2011). Hortelano et al. reported a 57‐year‐old male with known metastatic colorectal cancer, who suddenly developed bilateral parkinsonism. Computed tomography (CT) imaging revealed multiple brain metastases. The patient worsened rapidly, becoming akinetic and bedridden, and deceased 2 weeks after the manifestation of neurologic symptoms (Hortelano et al., 2010). Chang et al. described a 74‐year‐old male with renal cell carcinoma who later in the disease process exhibited bilateral bradykinesia, rigidity, and gait difficult. Cerebral metastases were found on CT (Chang et al., 2000). There is a wide agreement that patients with extracranial cancer who rapidly develop parkinsonian symptoms must be investigated for brain metastases in order to facilitate adequate therapy and improve disease prognosis (Chang et al., 2000; Hortelano et al., 2010; Ishihara et al., 2011).

#### 3.3.2 Paraneoplastic syndromes

PNSs are defined as a group of abnormal immune system reactions to neoplasms. PNS as a cause for parkinsonism is extremely rare. Topcular et al. discussed a 66‐year‐old male exhibiting bradykinesia, freezing of gait, rigidity, postural instability, and bradymimia, with a medical history of lung cancer with brain metastasis. CSF protein analysis revealed increased pattern 2 oligoclonal IgG bands, indicating probable neurologic PNS. Pulse steroid treatment was initiated with great success, and the patient's Unified Parkinson's Disease Rating Scale (UPDRS) score improved. The authors suggest that one must keep PNS parkinsonism in mind when presented with a patient quickly developing parkinsonism after a medical history of cancer, and that one should consider using steroids besides treating the underlying neoplasm if possible (Topcular et al., 2013). Golbe et al. reported a 42‐year‐old female presenting with weight loss, action tremor, and dysfunctional initiation of gait. Breast ductal adenocarcinoma metastatic to lymph nodes and liver was diagnosed a couple of months later and was treated with chemotherapy. The case was interpreted as probable paraneoplastic degeneration of the substantia nigra (Golbe et al., 1989). Li presented a similar case of a 39‐year‐old pregnant woman who exhibited tremor, facial stiffness, dysarthria, and reduced leg strength, who, when giving birth by cesarean section, was diagnosed with metastatic breast ductal adenocarcinoma in lymph nodes and the liver. There is currently no well‐developed pathophysiological model to explain how these cases of breast cancer cause PNS‐induced parkinsonism (Li, 2019). Parkinsonism has also been described secondary to PNS associated with the lymphoproliferative disorder lymphomatoid granulomatosis (Oliveras et al., 1988).

### 3.4 Parkinsonism induced by oncological treatment

#### 3.4.1 Radiotherapy

Reddy et al. described a 64‐year‐old male who was treated with radiotherapy for a mesencephalic low‐grade astrocytoma. One year after successful treatment, the patient developed unilateral hemiparkinsonism (Reddy et al., 2022). Franchino et al. compiled a series of case reports from a neurooncological database. One metastasis and six glioma patients, who all developed extrapyramidal symptoms not associated with tumoral mass effect or infiltration of the basal ganglia postirradiation, were included. Five of the patients quickly developed neurological symptoms, while two exhibited parkinsonism decades later, possibly due to idiopathic PD rather than secondary to radiotherapy. The disease progression was very slow or absent, which is inconsistent with idiopathic PD. Neurological disturbances observed in the affected patients included bradyphrenia, bradykinesia, hypomimia, dysfunctional gait, intermittent freezing, cognitive decline, apathy, and memory impairment. MRI with Fluid attenuated inversion recovery (FLAIR) revealed hyperintense regions within white matter. The levodopa response was weak to moderate (Franchino et al., 2019). Artusi et al. examined the medical history of a 61‐year‐old male who developed parkinsonism after radiotherapy for a grade 2 and 3 oligodendroglioma. Just like in the institutional series conducted by Franchino et al., MRI revealed white matter diffuse damage, which is commonly seen after radiotherapy. In agreement with Franchino et al., Artusi et al. interpreted this pathological change, even though there was no evidence of basal ganglia involvement, as the most likely cause to the observed parkinsonian symptomatology. Furthermore, the researchers hypothesized that potential pathologies in the larger cortico‐striato‐pallido‐thalamo‐cortical loop could explain this phenomenon (Artusi et al., 2015).

#### 3.4.2 Chemotherapy

Pereira et al. reported a 40‐year‐old female who developed bradykinesia and rigidity after unspecified chemotherapy. Persistent dopaminergic and anticholinergic treatment had desirable clinical effect. However, remission was not observed when trying to taper off medications, and the patient thus had to continue with initial therapy indefinitely. There was no need to increase dosages (Pereira et al., 2013). Other examples of parkinsonism emerging during chemotherapy include a patient who was also administered high‐dose metoclopramide for antiemetic purposes during the treatment (Markman et al., 1985), a dialysis‐supported patient who received high‐dose unspecified chemotherapy for multiple myeloma (Fleming & Mangino, 1997), and a patient who was treated with unspecified chemotherapy for non‐Hodgkin lymphoma (Howell & Sagar, 1994). Metoclopramide is a well‐known cause to drug‐induced parkinsonism.

#### 3.4.3 Oncological parkinsonism in children

It is extremely rare for idiopathic PD to develop in children, and this type, which is referred to as juvenile parkinsonism, is often associated with specific, high‐risk genes. Secondary parkinsonian symptoms in children following cancer treatment, although still uncommon, are often possible to prevent or reverse by optimizing or altering treatment. Furthermore, secondary parkinsonism after cancer therapy has been reported to be more common in general pediatric hospitals than rare genetic parkinsonian movement disorders (Pranzatelli et al., 1994). Cases of secondary parkinsonism in children have been described after craniospinal radiotherapy in both teenagers (Bernard & Chouinard, 2011; Voermans et al., 2006) and infants (Skiming et al., 2003), with somewhat varying responses to dopaminergic therapy. Other cases of secondary parkinsonism include an infant with acute leukemia who was treated with the chemotherapeutics vincristine and adriamycin (Boranic & Raci, 1979) and three children with refractory leukemia who were treated with bone marrow transplantation and high‐dose amphotericin B (Mott et al., 1995). Pranzatelli et al. reported the largest study so far on children with a spectrum of secondary parkinsonism, including six hospitalized children with parkinsonism. The symptoms of some of these patients were interpreted to be the result of cancer treatment, but the study contained a wide array of suggested etiologies in the different cases. All children improved dramatically, most having complete reversal of symptoms and none having to continue dopaminergic therapy (Pranzatelli et al., 1994).

### 3.5 Parkinsonism after tumor resection

There are numerous examples of parkinsonism developing after various types of tumor treatment, including surgical resection in the case of brain tumors. Ho et al. described a 36‐year‐old female who was surgically treated for a pituitary adenoma. A week after transsphenoidal surgery was performed, she developed hyponatremia as well as acute onset ataxia, hand tremor, and dysarthria. MRI indicated extrapontine myelinolysis, most likely because of too quick correction of the hyponatremia. The case was interpreted as parkinsonism, and this theory was strengthened by levodopa–carbidopa having an excellent effect. Neurological deficits improved gradually for months in association with her myelinolysis recovery, until complete reversal of symptoms was noted (Ho et al., 2019). Malomo et al. observed a 55‐year‐old male, who exhibited parkinsonism subsequent to the excision of a convexity meningioma, which was complicated by a massive postoperative epidural hematoma. Dopaminergic therapy resulted in slow but steady clinical improvement (Malomo & Emejulu, 2008). Wenning et al. reported a similar case of postoperative parkinsonism after the resection of a frontal meningioma (Wenning et al., 1999).

### 3.6 Treatment options for tumoral parkinsonism: The effect of dopaminergic therapy

#### 3.6.1 No effect

Eleven of the 25 (44%) studies initiating dopaminergic therapy on a patient suffering from some type of tumoral parkinsonism found no effect on clinical symptomatology (Alp et al., 2009; Bernard & Chouinard, 2011; Dolendo et al., 2003; Ertan et al., 2005; Golbe et al., 1989; Ho et al., 2008; Ishihara et al., 2011; Kleib et al., 2016; Li, 2019; Modreanu et al., 2020; Ruiz‐Escribano Menchen et al., 2020). In the case of Kleib et al., the patient had been taking levodopa for 4 months without effect, before MRI revealed a meningioma and surgery was performed (Kleib et al., 2016). Ho et al. prescribed levodopa and dopamine agonists, believing that the patient suffered from idiopathic PD, but due to the unresponsiveness of medication, brain CT was conducted and showed an astrocytoma (Ho et al., 2008). Modreanu et al. described a long process of levodopa adjustments in a patient with a known cavernoma. Initially, the patient was medicated with low‐dose levodopa, but the doses were gradually increased to 750 mg per day. However, the patient still claimed a lack of response, and this was later confirmed with negative levodopa and apomorphine tests. Levodopa treatment was discontinued, and no worsening of motor or nonmotor symptoms was observed (Modreanu et al., 2020). Alp et al. and Ertan et al. both reported patients with cavernomas, who refused surgical intervention and did not respond to levodopa even at high doses (Alp et al., 2009; Ertan et al., 2005). The growing teratoma syndrome with subsequent parkinsonism investigated by Dolendo et al. was managed with levodopa–carbidopa and amantadine, but to no avail (Dolendo et al., 2003). Levodopa was also unhelpful in the CNS lymphoma patient examined by Ishihara et al. (2011). Other cases where high doses of levodopa–carbidopa were ineffective in relieving parkinsonian symptoms include two patients with glioblastoma (Ruiz‐Escribano Menchen et al., 2020) and a teenager developing parkinsonism postradiotherapy (Bernard & Chouinard, 2011). Li discussed an unusual case of parkinsonian symptoms caused by breast ductal adenocarcinoma, who received levodopa to ease neurological deficits, but with no effect (Li, 2019). Yet another breast ductal adenocarcinoma leading to parkinsonian symptoms was illuminated by Golbe et al. This patient was treated with several drugs, including anticholinergics, baclofen, diazepam, levodopa–carbidopa, and plasmapheresis, all resulting in neither positive nor negative change in clinical presentation (Golbe et al., 1989). No studies included in this review have reported negative or unexpected side effects of dopaminergic treatment in patients suffering from tumoral parkinsonism.

#### 3.6.2 Low to moderate effect

Twelve of the 25 (48%) studies initiating dopaminergic therapy in individual patients suffering from some type of tumoral parkinsonism found partial effect on clinical symptomatology (Artusi et al., 2015; de Sèze et al., 1998; Dunbar et al., 2012; Franchino et al., 2019; Gherardi et al., 1985; Ho et al., 2019; Malomo & Emejulu, 2008; Pranzatelli et al., 1994; Reddy et al., 2022; Voermans et al., 2006; Wächter et al., 2011; Wimmer et al., 2020). Partial effect in this context is defined as low to moderate reversal of parkinsonians symptoms as a consequence of therapy with some sort of dopaminergic agent. Wimmer et al. wrote about a patient who was treated with levodopa and pramipexole for hemiparkinsonism associated with an arachnoid cyst. Although the treatment was effective at first, symptoms worsened over time as the underlying cysts progressed, thus leading to low treatment effect (Wimmer et al., 2020). De Sèze et al. described a case initially mistaken for idiopathic PD that was managed with 10 mg per day of selegiline and after half a year 300 mg of levodopa per day. Tremor and motor symptoms improved, but after a year the patient deteriorated, and radiological examination revealed an oligodendroglioma. Hence, oncological interventions replaced dopaminergic therapy (de Sèze et al., 1998). Ho et al. treated a rare case of acute extrapontine myelinolysis after pituitary adenoma resection with levodopa–carbidopa. Symptoms improved gradually over 2 months resulting in complete reversal of neurological deficits. Because of the rapid recovery, it is difficult to assess exactly what role antiparkinsonian medication played (Ho et al., 2019). Pranzatelli et al. similarly reported swift improvement in all study subjects in a cohort of six children with secondary parkinsonism. Although diphenhydramine, benztropine, amantadine, and levodopa–carbidopa with or without selegiline were utilized, it is yet again difficult to determine how important the dopaminergic therapy was for recovery (Pranzatelli et al., 1994). Wächter et al. reported moderate levodopa response in a patient with thalamic butterfly astrocytoma (Wächter et al., 2011). Gherardi et al. also concluded that levodopa initially had moderate effects, although in a primary CNS lymphoma (Gherardi et al., 1985). In both cases, the symptoms gradually worsened, and this motivated neuroimaging and subsequent findings of brain neoplasms. This chain of initial low to moderate effect of dopaminergic therapy in what is thought to be idiopathic PD, followed by unusually rapid exacerbation in clinical presentation, and finally neuroimaging and the discovery of an unknown brain tumor, is a recurring pattern in several tumoral parkinsonism case reports (de Sèze et al., 1998; Gherardi et al., 1985; Wächter et al., 2011). Several studies have described low to moderate effect of dopaminergic therapy in parkinsonism related to iatrogenic effects of tumor treatment following surgery (Malomo & Emejulu, 2008) and radiotherapy (Artusi et al., 2015; Franchino et al., 2019; Reddy et al., 2022; Voermans et al., 2006). Dunbar et al. investigated the potential use of basal ganglia drugs as palliative medication in a cohort of 21 patients with late‐stage brain tumors. They found that agents such as methylphenidate, modafinil, levodopa, and amantadine relieve at least one major parkinsonian symptom in 86% of the included patients, thus indicating that these drugs could have utility in palliative care of brain cancer patients with parkinsonism (Dunbar et al., 2012).

#### 3.6.3 Excellent effect

Two of the 25 (8%) studies initiating dopaminergic therapy on patients suffering from some type of tumoral parkinsonism found excellent effect on clinical symptomatology (Pereira et al., 2013; Straube & Sigel, 1988). Excellent effect in this context is defined as complete reversal of parkinsonian symptoms as a consequence of therapy with some sort of dopaminergic agent. Straube et al. treated a low‐grade glioma patient, presenting with resting tremor, rigidity, akinesia, and slight right leg paresis, with 400 mg of levodopa–carbidopa and 12.5 mg of bromocriptine per day. Complete reversal of symptoms was observed immediately after treatment initiation, and there had been no deterioration 5 years after onset, without altering dosages. The slight right leg paresis did not regress nor progress during this time (Straube & Sigel, 1988). Pereira et al. investigated three middle‐aged female patients who developed parkinsonism secondary to oncological treatment, more specifically systematic chemotherapy for lymphoma, whole‐brain radiotherapy for glioma, and motor cortex radiosurgery for pulmonary adenocarcinoma metastasis, respectively. Parkinsonian symptoms started after 1 year of oncological intervention and consisted of mild bradykinesia and rigidity in all cases. Additionally, one patient suffered from moderate tremor. Anticholinergic and dopaminergic therapy had excellent results in all cases, and none of the patients had to increase dopaminergic treatment 2–5 years after onset. However, none of the patients had reversal of symptoms without medication and required persistent antiparkinsonian therapy (Pereira et al., 2013). Both these studies described full symptomatic reversal with dopaminergic therapy, but insufficient clinical presentation without treatment.

## 4 DISCUSSION

One key difference between idiopathic PD and secondary parkinsonism is that idiopathic PD typically responds better to pharmacological dopaminergic treatment compared to secondary parkinsonism (Rizek et al., 2016). However, based on the results in this review, this might not always hold true for tumoral parkinsonism. Most guidelines agree that an MRI or CT should always be a part of PD diagnostics (Arcaya Navarro et al., 1986; Choi et al., 2012; Cicarelli et al., 1999; Ho et al., 2008; Wächter et al., 2011). Furthermore, a tumor patient might also have idiopathic PD. Therefore, methods such as MIBG myocardial scintigraphy or DaTSCAN should be considered for further differentiation (Ogawa et al., 2018). It should, however, be pointed out that dopamine transporter imaging has been pathological in single cases clinically identified as tumoral parkinsonism (Modreanu et al., 2020). Because the tumor can affect various brain segments, patients with tumoral parkinsonism seldom solely manifest with parkinsonian symptoms. Classic symptoms, such as headaches, seizures, and behavioral changes, typically accompany motor symptoms (Perkins & Liu, 2016). Furthermore, the parkinsonian clinical picture in tumor cases can vary widely and is often atypical compared to idiopathic PD (Saleh et al., 2021).

Although brain metastases outnumber primary brain tumors by a ratio of 10:1 and occur in as much as 25% of cancer patients (Saha et al., 2013), metastases have rarely been reported to cause tumoral parkinsonism. This discrepancy might exist because brain metastases of cancer with extracranial origin usually develop in the later stages of neoplastic disease when other neurological deficits also manifest, thus confounding symptoms. There was no major difference in symptomatology between brain metastasis and primary brain tumor patients. The general pattern across all these cases is that there is a lot of uncertainty as to how the lesion causes parkinsonian symptomatology. Several explanatory theories have been proposed, including distant mass effect, vascular dysfunction, and nigrostriatal influence, but most studies have not been able to motivate these speculative pathophysiological causalities.

PNSs are complex abnormal immunological reactions to neoplasms and have been reported to cause secondary parkinsonism (Golbe et al., 1989; Li, 2019; Oliveras et al., 1988; Topcular et al., 2013). Recent research indicates that these immune mechanisms may also affect the pathogenesis in idiopathic PD (Chaná‐Cuevas et al., 2020; Xing et al., 2022), as microglia, T‐cells, and cytokines have been shown to be abnormally increased in these patients (Rocha et al., 2018; Xing et al., 2022). Although not included in the systematic search, Stiff person syndrome deserves to be mentioned in this immunological context. It is a rare autoimmune movement disorder, clinically characterized by fluctuating rigidity and stiffness of the axial and proximal lower limb muscles, with superimposed painful spasms and continuous motor unit activity on electromyography. In 5% of patients with Stiff person syndrome, the underlying cause is PNS. So forth, PNS can cause several movement disorders including parkinsonism and Stiff person syndrome (Hadavi et al., 2011). This connection between paramalignancy, autoimmunity, antibodies, and movement disorders is still heavily underexplored and demands more attention. Oncological treatment has been reported to cause parkinsonism in both adults and children (Artusi et al., 2015; Pranzatelli et al., 1994; Wenning et al., 1999), and while it has been hypothesized that white matter diffuse damage related to radiotherapy might cause parkinsonism (Franchino et al., 2019), the biological mechanisms at play are still not well understood.

Of the total 25 studies, 44% reported no effect, 48% low to moderate effect, and 8% excellent effect on parkinsonian symptoms after trying some sort of dopaminergic therapy (see Table 3 for details). This indicates that, on the contrary to popular belief, dopaminergic therapy might indeed have potential to relieve symptoms in cases of tumoral parkinsonism. However, the selected analysis approach is gross and simple, and cannot safely determine that there is indeed clinical benefit with dopaminergic treatment. If dopaminergic treatment was indeed to improve symptoms in brain tumor patients with secondary parkinsonism, this would be beneficial to the quality of life of the patient.

**TABLE 3:** The impact of dopaminergic treatment on tumoral parkinsonism symptomatology categorized by study, level of effect, prescribed medication, number of patients, and cause of parkinsonism.

Study (Name. Title.)	Effect of dopaminergic treatment	Dopaminergic medication used, dosage if reported (time if reported)	Patients included	Type of tumoral parkinsonism
Kleib et al. Hemiparkinsonism secondary to sphenoid wing meningioma.	None	Levodopa (4 months)	1	Meningioma
Ho et al. Hemiparkinsonism secondary to an infiltrative astrocytoma.	None	Levodopa + a dopamine agonist	1	Astrocytoma
Modreanu et al. Nine‐years follow‐up of cavernoma located in basal ganglia mimicking Parkinson's disease.	None	Levodopa, low dose increased to 750 mg daily	1	Cavernoma
Alp et al. Cavernous hemangioma: a rare cause for secondary parkinsonism: a case report.	None	Levodopa	1	Cavernoma
Ertan et al. Parkinsonism caused by cavernoma located in basal ganglion.	None	Levodopa, high‐dose	1	Cavernoma
Dolendo et al. Parkinsonism as an unusual presenting symptom of pineal gland teratoma.	None	Levodopa–carbidopa + amantadine	1	Growing teratoma syndrome
Li. Secondary Parkinson disease caused by breast cancer during pregnancy: A case report.	None	Levodopa–benserazide	1	Paraneoplastic syndrome, breast adenocarcinoma
Golbe et al. Paraneoplastic degeneration of the substantia nigra with dystonia and parkinsonism.	None	Levodopa–carbidopa	1	Paraneoplastic syndrome, breast adenocarcinoma
Ishihara et al. Clinicoradiological changes of brain NK/T cell lymphoma manifesting pure akinesia: a case report.	None	Levodopa 300 mg/day + carbidopa 30 mg/day	1	Metastasis of natural killer/T‐cell lymphoma
Ruiz‐Escribano Menchen et al. 2 clinical cases of secondary parkinsonism due to cerebral glioblastoma.	None	Levodopa	2	Glioblastoma
Bernard et al. A unique pediatric case of radiation‐induced parkinsonism.	None	Levodopa–carbidopa, high‐dose	1	Oncological parkinsonism in children
Wimmer et al. Symptomatic hemiparkinsonism due to extensive middle and posterior fossa arachnoid cyst: case report.	Partial	Levodopa, max 1200 mg/day + pramipexole	1	Arachnoid cyst
De Sèze et al. Hemiparkinsonism revealing an infiltrating low‐grade oligodendroglial tumor.	Partial	Selegiline, 10 mg/day—6 months after replaced by levodopa, 300 mg/day	1	Low‐grade oligodendroglioma
Ho et al. Acute parkinsonism as an unexpected consequence of pituitary adenoma resection: A case report.	Partial	Levodopa–carbidopa	1	Postresection
Voermans et al. Secondary parkinsonism in childhood: A rare complication after radiotherapy.	Partial	Dopamine agonists	1	Oncological parkinsonism in children
Malomo et al. Could Parkinsonism complicate craniotomy and excision of convexity meningioma? A case report.	Partial	Levodopa	1	Postresection
Reddy et al. Parkinson's Syndrome After Cranial Radiotherapy: A Case Report.	Partial	Levodopa–carbidopa	1	Postradiotherapy
Wächter et al. Slowly progressive Parkinson syndrome due to thalamic butterfly astrocytoma.	Partial	Levodopa	1	Astrocytoma
Gherardi et al. Parkinsonian syndrome and central nervous system lymphoma involving the substantia nigra. A case report.	Partial	Levodopa	1	Primary CNS lymphoma
Franchino et al. Post‐irradiation parkinsonism: An institutional series.	Partial	Levodopa	7	Postradiotherapy
Artusi et al. Actinic Parkinsonism: A case report.	Partial	Levodopa, low‐dose	1	Postradiotherapy
Dunbar et al. The use of basal ganglia drugs as palliative therapy for secondary parkinsonism in malignant CNS tumor patients: A University of Florida experience.	Partial	Methylphenidate, modafinil, levodopa, amantadine	21	Malignant glioma
Pranzatelli et al. Clinical spectrum of secondary parkinsonism in childhood: A reversible disorder.	Partial	Amantadine, levodopa‐carbidopa with/without selegiline, diphenhydramine, benztropine	6	Oncological parkinsonism in children
Straube et al. Parkinsonian syndrome caused by a tumour of the left supplementary motor area.	Excellent	Levodopa–carbidopa, 400 mg/day + bromocriptine, 12.5 mg/day	1	Low‐grade glioma
Pereira et al. Persistent parkinsonian syndrome induced by radiotherapy and chemotherapy.	Excellent	Levodopa + anticholinergics	3	Postchemotherapy

Almost all studies included in this review have a major limitation: they neglect nonmotor symptoms. Nonmotor symptoms affect the quality of life immensely (Prakash et al., 2016) and deserve more recognition. Because the included articles do not report sufficient nonmotor data, discussing symptoms in this review, including the fact that dopaminergic treatment might ease them, almost exclusively refers to motor symptoms when not stated otherwise. Since patients with a brain tumor often experience anxiety and depression, there is significant overlap with nonmotor symptoms potentially related to the secondary parkinsonism. The authors therefore suggest that it is important to recognize nonmotor symptoms, and based on clinical experience, that one could consider dopaminergic therapy in tumoral parkinsonism patients with mixed nonmotor symptoms, for example, for improving depression, anxiety, or daytime sleepiness.

### 4.1 Recommended dopaminergic therapy and dosage

Most studies in which dopaminergic treatment was initiated used levodopa (Table 3). Levodopa has likely been the firsthand therapeutic choice for tumoral parkinsonism because of its long history as the most efficacious drug in the treatment of parkinsonism and its relatively few side effects. Several dosages of levodopa have been tried (Table 3), but the data in these studies are not sufficient to make sound conclusions regarding dosage. Treatment results based on specific anatomical location and histological entity are inconsistent and limited. However, cavernomas and adenocarcinoma PNSs might respond poorly, and parkinsonism induced by oncological treatment and brain resection well, to levodopa (Table 3). For the time being, it can probably therefore be recommended that, just like in atypical parkinsonism (Fanciulli et al., 2019), one may increase the dosage of levodopa slowly to a maximum of 1000 mg per day in tumoral parkinsonism before assessing therapeutic effect. Dopamine agonists and amantadine could also be considered, either in addition to or as an alternative to levodopa. In the presence of nonmotor symptoms like depression and anxiety, dopaminergic therapy could be tried in addition to the standard treatment.

### 4.2 Limitations of the study

This study regarding secondary tumoral parkinsonism was conducted as a systematic literature review. Articles included were collected through the databases PubMed and Embase. Subject headings such as Emtree terms and MeSH terms were utilized when feasible. Otherwise, free search terms were used, having the additional advantage of finding not‐yet‐tagged articles. Using the term “astrocytoma” instead of the broader category “glioma” narrowed the search. A majority of the studies did not compare to controls, lacked randomization and blinding, reported single cases, or had small cohorts. Several articles did not report full information regarding which drugs had been used or in what dosage, thus limiting the capacity to analyze the effect of dopaminergic treatment as visualized in Table 3, as well as the results section reporting on chemotherapy.

## 5 CONCLUSION

The results of this study suggest that several brain neoplasms, PNSs, and oncological treatments can cause parkinsonism, but that the diverse pathophysiology remains to be fully understood. Published studies indicate that dopaminergic therapy might have relieving effects on motor symptomatology in patients with tumoral parkinsonism. Dopaminergic therapy has been shown to have a substantial impact on patient quality of life in several cases and should therefore be considered in patients with suspected or confirmed tumoral parkinsonism.

## AUTHOR CONTRIBUTIONS

**Gustav Cedergren Weber**: Research project (conception, organization, and execution); statistical analysis (design and execution); manuscript (writing of the first draft, and review and critique). **Jonathan Timpka**: Research project (conception, organization, and execution); statistical analysis (design, and review and critique); manuscript (writing of the first draft, and review and critique). **Anna Rydelius**: Research project (conception); statistical analysis (review and critique); manuscript (review and critique). **Johan Bengzon**: Research project (conception); statistical analysis (review and critique); manuscript (review and critique). **Per Odin**: Research project (conception, organization, and execution); statistical analysis (design, and review and critique); manuscript (review and critique).

## CONFLICT OF INTEREST STATEMENT

The authors declare no conflicts of interest.

## FUNDING INFORMATION

No specific funding was received for this work.

### PEER REVIEW

The peer review history for this article is available at https://publons.com/publon/10.1002/brb3.3151.

## Data Availability

The data that support the findings of this study are available on request from the corresponding author. The data are not publicly available due to privacy or ethical restrictions.
